# A new species of frog-biting midge from Papua New Guinea with a key to the described Corethrellidae of the Australopapuan region (Diptera, Corethrellidae, *Corethrella*)

**DOI:** 10.3897/zookeys.795.28543

**Published:** 2018-11-05

**Authors:** Gunnar Mikalsen Kvifte, Ximena E. Bernal

**Affiliations:** 1 Department of Biological Sciences, Purdue University, 913 West State Street, IN-47906 West Lafayette, USA Purdue University West Lafayette United States of America; 2 Smithsonian Tropical Research Institute, Apartado 0843-03092, Balboa, Ancón, Panamá, República de Panamá Smithsonian Tropical Research Institute Balboa Panama

**Keywords:** Culicoidea, Culicomorpha, Papua New Guinea, taxonomy

## Abstract

*Corethrellaoppositophila* Kvifte & Bernal, **sp. n.** is described based on one male and six female specimens collected at 2200 m a.s.l. on Mount Wilhelm, Papua New Guinea. The species is the fourth species of frog-biting midge described from this country and appears similar to *Corethrellasolomonis* Belkin based on pigmentation of legs and abdominal tergites. It differs from *C.solomonis*, however, in the shape of female flagellomeres I–III, and in the thorax which has a dark brown vertical stripe. The new species is named for its sexually dimorphic flagellomeres, which are short and squat in the female and elongate in the male. These differences in morphological characters are discussed in light of the likely sexual differences in functional uses of the antennae, as males use them for mating only whereas females use them both for mating and prey location. An emended key is presented to the described Australopapuan species of Corethrellidae.

## Introduction

Frog-biting midges (Diptera: Corethrellidae; *Corethrella* Coquillett) are widespread in the tropics, noteworthy in their females being specialized micropredators of frogs and toads. Locating their hosts primarily by auditory cues (Borkent 2008, Bernal and de Silva 2015), they are an emerging study system in studying the ecology and evolution of acoustic eavesdropping behaviour (Bernal et al. 2006, Grafe et al. 2008, 2018, de Silva et al. 2015, Caldart et al. 2016, Legett et al. 2018). Their fossil record extends back to the early Cretaceous and their ancient age is reflected in their biogeography which suggests Gondwanan vicariate events (Borkent 2008, Baranov et al. 2016). Including the new species described herein, 110 species are known from the extant fauna; however, this family has received little taxonomic study across their broad worldwide distribution (Borkent 2008, Borkent and Grafe 2012, Fei-peng et al. 2015, Baranov et al. 2016, Caldart et al. 2016).

In particular, the frog-biting midges from the Afrotropical, Oriental, and Australasian regions have received little attention apart from limited regional treatments (Colless 1986, Borkent and Grafe 2012). Insufficient information from these biogeographic regions has ultimately hampered our understanding of this group’s phylogeny and evolutionary history (Borkent 2008). Since the three earliest diverging extant lineages of frog-biting midges occur in Australasia (Borkent 2008, Baranov et al. 2016), lack of material from this region is particularly problematic. Only eleven species have been recorded from this region: three from Papua New Guinea, five from Australia, one from New Zealand, and two from the Solomon Islands. The rich tropical rainforests of the Australopapuan region are, however, likely to harbour much higher species diversity than currently recognized, as evident from the low number of specimens known for the described species (see discussion in Borkent 2008). Species richness comparable to those of Borneo or Costa Rica (Borkent 2008, Borkent and Grafe 2012), if not even higher, is to be expected in the Australopapuan region.

In this study, we examine Malaise trap material collected by Leponce et al. (2016) and describe *Corethrellaoppositophila* Kvifte & Bernal, sp. n. based on an adult male and six adult females. This species is similar to *C.solomonis* Belkin, 1962 from the Solomon Islands, for which only the female has been described. The discovery of a male of this putative group of Australasian and Oceanic species could thus add phylogenetic information. We also provide a key to the frog-biting midges of the Australopapuan region. Finally, we reveal interesting sexual dimorphism in the antenna and discuss how these morphological characters may relate to their different functions in each sex.

## Materials and methods

Specimens were collected in Malaise traps at Mount Wilhelm, Morobe province, in October 2012, as described by Leponce et al. (2016). All specimens were stored in 96% alcohol in the Royal Belgian Institute of Sciences (**RBINS**). Prior to examination the specimens were cleared in 10% KOH at room temperature and mounted on slides in Canada balsam following methods outlined in Kirk-Spriggs (2017). Observations were performed using a Nikon Eclipse Ci compound microscope. Measurements were made using an ocular micrometer and are given in μm with an accuracy of +/- 2.5 μm, except wing length which is given in mm with an accuracy of 0.01 mm. When three or more specimens were available, measurements are given as ranges followed by means in parentheses. Photographs were taken with a Nikon DS-Fi2 camera and line drawings were prepared using a drawing tube from a Leitz Diaplan compound microscope attached to the Nikon Eclipse Ci.

Morphological characters and terminology mainly follows Borkent (2008). Additional characters, however, were also examined. The eyebridge, for instance, is of high taxonomic utility in Psychodinae, and both its width in facet rows and median extent are used here (Kvifte and Wagner 2017). The inner head skeleton of Corethrellidae, specifically the cibarium and tentorium appear to show some interspecific variation; terminology for these provisionally follows McAlpine (1981) and are labelled in figures 2a–b. Some chaetotaxic characters of the male and female postabdomen are also included: for males, the width/length proportions of tergite 8 and the width of the median hairless stripe on tergite 9 and, for females, the specialized setae on the proctiger.

### Corethrella oppositophila

Taxon classificationAnimaliaDipteraCorethrellidae

Kvifte & Bernal sp. n.

http://zoobank.org/DB8CE9C3-BFA2-4560-9FE6-D2C2BB17478A

Figs 1 , 2

#### Type material.

Holotype male. PAPUA NEW GUINEA: Morobe province, Mount Wilhelm, 5.758978°S, 145.18607°E, 2200 m a.s.l., 27.X.2012, leg. Mogia, Lilip, Vohotny & Leponce (Malaise trap). Six paratype females, same locality as holotype but collection dates 17.X.2012, 22.X.2012, 25.X.2012, 28.X.2012, and 31.X.2012 (two specimens). All specimens in collections of RBINS.

#### Diagnosis.

Only extant species of Corethrellidae with the following combination of characters: wing with a mid-length band of dark pigmentation and scales, thorax brown with anterior two thirds of scutum, prosternum and katepisternum light brown, abdominal tergites light brown with anterior dark bands, dorsomedial seta of male gonocoxite parallel with proximalmost seta in dorsal row.

#### Description.

**Figure 1.**
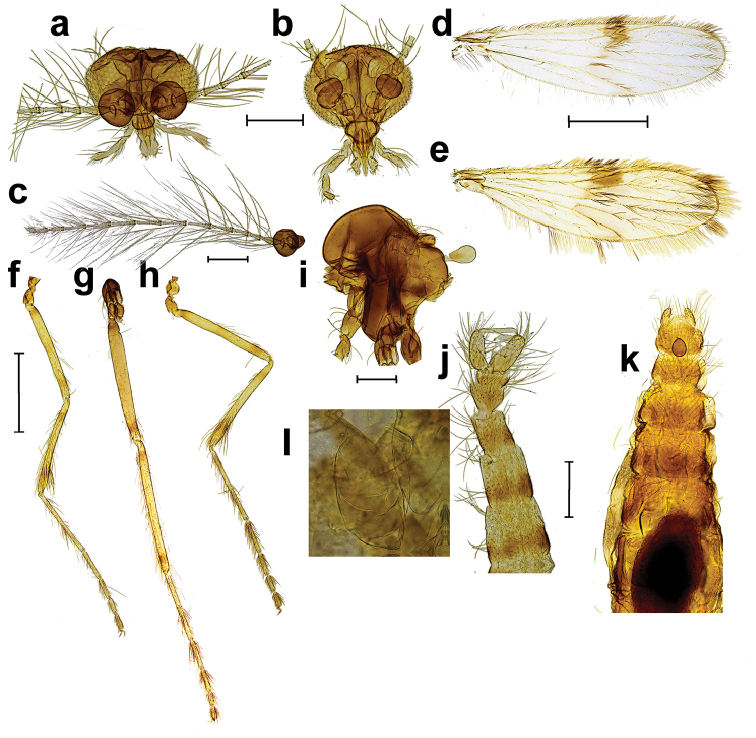
*Corethrellaoppositophila* Kvifte & Bernal, sp. n., male and female **a** male head **b** female head **c** male antenna **d** male wing **e** female wing **f** hind leg of female **g** mid leg of female **h** fore leg of female **i** thorax of female **j** male abdomen **k** female abdomen with blood meal **l** egg in female abdomen. Scale bars: 200 μm (**a, b, c, i, j, k**); 500 μm (**d–g**). Views: frontal (**a, b**), dorsal (**d, e, j–l**), posterior (**f–h**).

**Figure 2.**
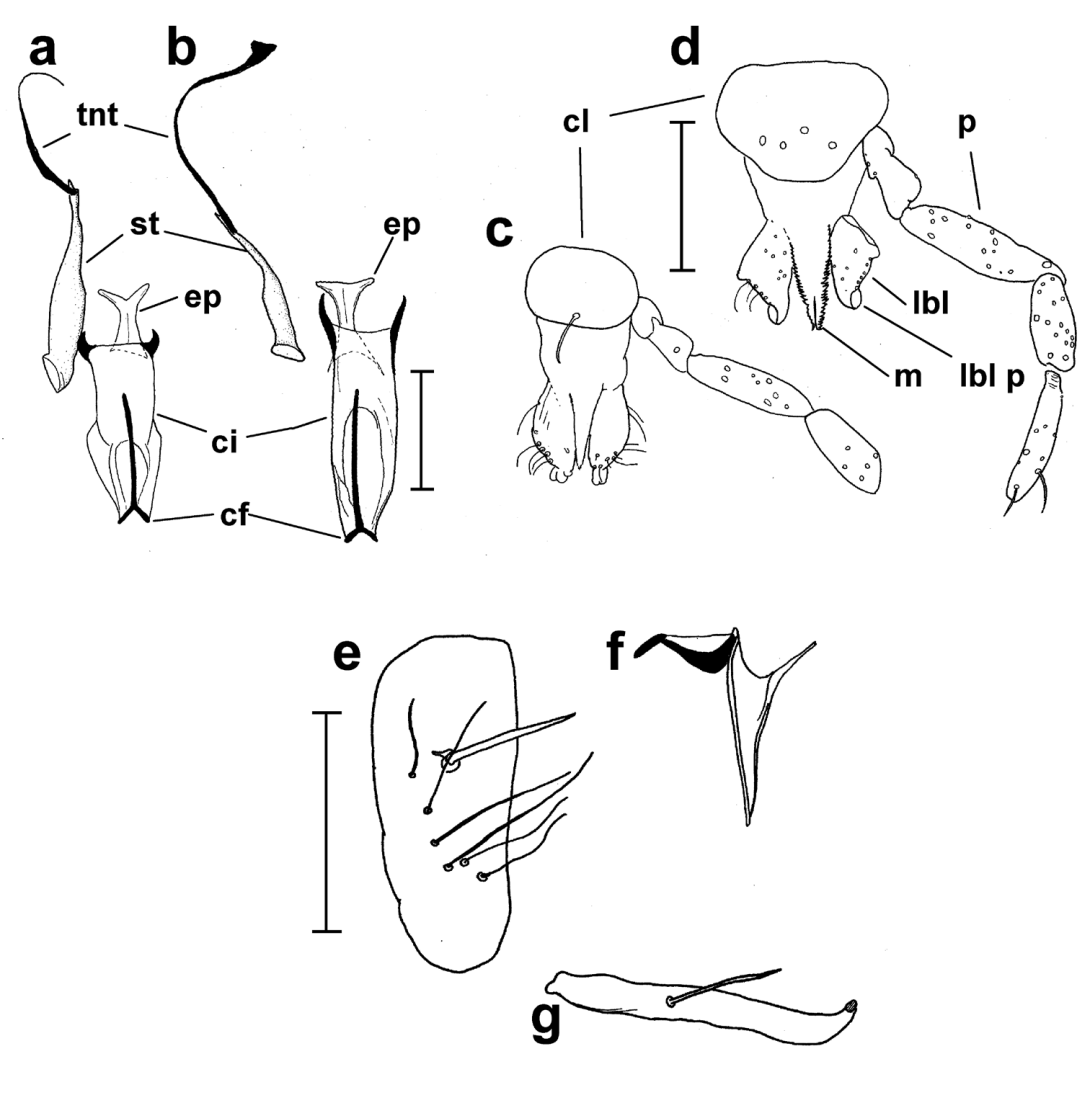
*Corethrellaoppositophila* Kvifte & Bernal, sp. n., male and female **a** male hypopharynx, cibarium and stipes **b** female hypopharynx, cibarium and stipes **c** male clypeus and mouthparts **d** female clypeus and mouthparts **e** gonocoxite **f** paramere and aedeagus (only one paramere shown) **g** gonostylus. Scale bars 80 μm (**a, b**); 100 μm (**c–g**). Abbreviations: cf – cibarial fork, ci – cibarium, cl – clypeus, ep – epipharynx, lbl – labellum, lbl – labellar projection, m – mandible, p – palpal segment III, st – stipes, tnt – tentorium. Views: frontal (**a–d**), dorsal (**e–g**).

Adult male (n = 1). ***Head*** (Figs 1a, 2a, 2c) broader than long. Eyebridge of five rows of facets, constricting towards median. Four frontal setae present. Antenna (Figure 1c) with pedicel dark brown, scape and flagellum paler. Pedicel without setae longer than length of pedicel. Length of flagellomeres 120, 62.5, 62.5, 85, 115, 132.5, 132.5, 130, 130, 125, 117.5, 82.5, 65, 80, terminal flagellomere bifurcate. Three sensilla coeloconica on flagellomere I, one sensillum coeloconicum on each of flagellomeres IX–XIII. Palpus uniformly pale brown with segment III of uniform width or slightly broader at mid length, length of palpal segments 37.5, 42.5, 100, 77.5, N/A (5^th^ palpal segments missing in specimen). Palpal segment I with single lateral elongate seta, segment II with two elongate setae, and one short. Clypeus broadly oval with single medial seta. Labellum oval. Cibarial pump, hypopharynx, tentorium and stipes as in Figure 2a.

***Thorax*** brown with anterior two thirds of scutum, prosternum and katepisternum light brown. Dorsocentral row without elongate setae at posterior end. Prescutal suture narrow, extending more than two thirds of way to dorsocentral row. Anterior anepisternum divided diagonally into dorsal and ventral portions, dorsal portion about twice as large as ventral; posterior anepisternum undivided, posterior half without distinct setae. Haltere paler than thorax.

***Wing*** (Figure 1d) 1.75 mm long, 0.48 mm wide, R_1_ 1.31 mm long. Apex of R_2_ at level with M_1_. Membrane with patch of dark infuscation from Sc to stem of R_2+3_, paler infuscation present over crossveins r-m and m-cu. Midlength and subapical bands of pigmented scales present. Wing scales narrow, those on C nearly twice as wide as those on other veins.

***Legs*** light brown with rings of darker pigmentations basally and subbasally on all femora and tibiae, more indistinct on midtibia. Fore- and midtarsi with banding. With only slender setae, lacking scales. Claws on fore- and midlegs unequal, hind leg claws equal, all simple, without basal prongs or empodia. Ratio of foreleg Ta_3_/Ta_4_ = 1.56.

*Abdomen* (Figure 1j) Light brown with darker brown mottled bands anteriorly on each tergite, sternites I–II pale, other sternites light brown with darker brown mottled bands anteriorly. Tergites and sternites VIII and IX light brown; length of segment VIII 112.5, distally 2.5 times as wide as base; hairless stripe medially on tergite IX 35 μm wide.

*Genitalia* (Figure 2e–g). Gonocoxite uniformly pale brown, tapering gently towards apex; all setae of similar length; with well-defined dorsal row of six setae of uniform length and thickness. Dorsomedial seta stout, tapering from non-expanded base. Gonostylus sinuous, of equal thickness except tapering apically, one elongate, thick subbasal seta situated on inner surface (ventrally), with thick, blunt subapical peg; subbasal seta 0.4 length of gonostylus. Parameres comprised of a sclerotized S-shaped part and a less weakly sclerotized egg-shaped part. Aedeagus slender, tapering gradually to apex, reaching beyond dorsomedial seta, lateral margins meeting apically.

Adult female (n = 6) As for male, with following differences. *Head* (Figure 1b) Eyebridge of 5–6 rows of facets, constricting towards median. Coronal suture long, extending ventrally to between antennal bases. All available specimens with flagellum broken, length of preserved flagellomeres (n = 4) 70–80 (74), 42.5–45 (43), 45–50 (47), first flagellomere with three sensilla coeloconica. Length of palpal segments (n = 6, 6, 6, 4, 2) 35–50 (41), 40–47.5 (45), 87.5–97.5 (93), 70–80 (76), 80–95 . Clypeus broadly hexagonal, with anterior margin about half length of posterior margin,, with 1–5 setae in single row. Mandibular teeth small, pointed. Labellum rectangular with apicomedial projection. Cibarial pump, hypopharynx, tentorium, and stipes as in figure 2b.

*Thorax* (Figure 1i) brown with anterior third of scutum, prosternum, mediotergite, metaepisternum, scutellum, and metakatepisternum light brown.

*Wing* (Figure 1e) 1.73–2.00 (1.79) mm long, 0.46–0.60 (0.53) mm wide. R_1_ 1.19–1.35 (1.24) mm long.

*Legs* (Figure 1f–1h) Claws of each legs equal to those of others, equal on each leg, simple, with empodia slender, feather-shaped. Ratio of foreleg Ta_3_ / Ta_4_ = 1.35

*Genitalia* (Figure 1k) with 2–6 microseta subapically on proctiger.

*Egg* (n = 15, Figure 1I) length 240, width 127.5 mm.

#### Biology.

Females have biting mouthparts and one paratype was collected with blood in its gut (Figure 1k). Another paratype female was preserved with 15 eggs in her abdomen; these were not preserved well enough, however, to allow morphological comparison with other described Corethrellidae eggs.

#### Distribution.

Known only from the type locality on Papua New Guinea, where it was collected in a Malaise trap at 2200 m.a.s.l.

#### Etymology.

From Latin *opposita*, opposite, and Greek φίλος *(philos*), friend. “Opposites attract” – referring to the stark sexual dimorphism of the basal flagellomeres of the male and female antennae.

#### Remarks.

The new species keys to *C.solomonis* in Borkent (2008) but differs from that species by its thorax being more extensively brown (see description above and compare with Borkent 2008: fig. 38B) and having much shorter flagellomeres in the female. The male of *C.solomonis* is unknown.

The male and females of *C.oppositophila* Kvifte & Bernal sp. n. have been associated based on similarity of pigmentation, together with co-occurrence in the same Malaise trap at the same time.

##### Key to the described Corethrellidae of the Australopapuan region

**Table:** 

1	Wings without pigmented markings	**2**
–	Wings with pigmented bands	**3**
2	Wing veins largely without scales. Papua New Guinea	***C.evenhuisi* Borkent, 2008**
–	Wing veins with scales. Papua New Guinea	***C.pauciseta* Borkent, 2008**
3	At least hind tibia with dark band at base	**4**
–	Tibiae unicolorous or with well-defined dark bands only at apices	**9**
4	Abdominal tergites uniformly pigmented. Hind tibia with dark band at base	**5**
–	Abdominal tergites with dark bands anteriorly. All tibiae with dark bands at base and subapically	**6**
5	Wing with dark scales restricted to mid-length band and apical margin. Solomon Islands	***C.canningsi* Borkent, 2008**
–	Wing with two bands of dark scales in addition to apical patch. Papua New Guinea	***C.varia* Borkent, 2008**
6	Femora apically with pigmented dark band (fig. 1f–1h)	**7**
–	Femora apically pale. Queensland (Australia)	***C.mckeeveri* Colless, 1986**
7	Thorax with pleura pale with narrow brown stripes. Solomon Islands	***C.solomonis* Belkin, 1962**
–	Thorax with pleura mostly brown.	**8**
8	Wing with unicolorous scales on anterior margin. Thorax with some pale mottling on pleura, scutum unicolorous brown. Australia	***C.collessi* Borkent, 2008**
–	Wing with contrasting, i.e. both darker and lighter, scales on anterior margin. Thorax without mottling on pleura, scutum lighter on anterior two thirds. Papua New Guinea	***C.oppositophila* Kvifte & Bernal, sp. n.**
9	Abdominal tergites I–VII with dark pigmentation only apicolaterally, sternites completely pigmented. Tibiae without dark bands. New Zealand	***C.novaezealandiae* Tonnoir, 1927**
–	Abdominal tergites I–VII with entire hind margin darkly pigmented, sternites with incomplete pigmentation. Tibiae with apical dark bands. Australia	**10**
10	Thorax dark brown. Wing with three distinct bands of pigmentation	***C.marksae* Colless, 1986**
–	Thorax light brown. Wing with pigmentation restricted to single dark midlength band	**11**
11	Head wider than long. Flagellomere I about four times as long as wide	***C.pallidula* Bugledich, 1999**
–	Head circular in frontal view. Flagellomere I about twice as long as wide	***C.alba* Borkent, 2008**

## Discussion

Apart from its darker thorax and shorter female flagellomeres, the female of *C.oppositophila* resembles *C.solomonis*. Because of the overall similarity and geographical proximity of these two species, we consider it likely that these species are closely related, and possibly forming a group with *C.mckeeveri* Colless, 1994 as suggested by Borkent (2008) and Baranov et al. (2016).There is, however, currently no robust synapomorphic evidence available to support this group. These three species all have many plesiomorphic characters and are among the earliest diverging lineages of Corethrellidae, only the Australian *C.marksae* species group and the New Zealandian *C.novaezealandiae* are earlier divergences within the family (Borkent 2008, Baranov et al. 2016).

The male of *C.oppositophila* is similar to that of *C.mckeeveri* both in the shape of the parameres and the presence of an apical peg on the gonostylus, characters that also appear to be similar to those observed in *C.marksae* Colless, 1986. It can be separated from both of these species based on the gonocoxite having its dorsomedial setae on level with the proximalmost seta of the dorsomedial row, thus resembling the Afrotropical *C.harrisoni* Freeman, 1962.

Males and females of *C.oppositophila* appear to have highly pronounced sexual dimorphism in the length and width of the basal flagellomeres. These flagellomeres are elongate in the observed male, whereas in all observed females they are short and bead-shaped (Figure 1a–c). We deem it likely that these striking morphological differences translate into sex-specific adaptations to detect different acoustic cues and signals: in the one Corethrellidae species where courtship has been studied the most comprehensively, both sexes use sound in mate recognition (de Silva et al. 2015). Females in addition use auditory cues in locating frogs on which to feed, meaning their antennae have to respond to a broader range of acoustic stimuli than those of the males. Borkent (2008) showed that shortened basal flagellomeres in the female are apomorphic in Corethrellidae within Culicoidea, and that different conditions of basal flagellomere shortening have arisen on multiple occasions in frog-biting midges (characters 37, 59 and 77 in Borkent 2008, p. 216, 221 and 225). Judging from illustrations in Borkent (2008), however, sexual dimorphism in flagellomere length is not universal within the group, and even in the early diverging Australopapuan lineages there is considerable variation. More data are needed to ascertain how widespread these sexual dimorphisms are and how these morphological differences are related to the putative functional differences in both sexes as outlined above.

The material from which the new species was identified stems from the inventory project of Leponce et al. (2016), which sampled along the entire 3200 m elevational gradient of Mount Wilhelm, Papua New Guinea. For the present study, only material from 1200–3200 m a.s.l. was available for examination, and frog-biting midges were only present at the sampling station at 2200 m elevation. This observation is unlikely to reflect any real absence of *Corethrella* at lower elevations in Papua New Guinea, however, as the three previously described species from this island have been collected at 122, 1250, and 1300 m elevation (Borkent 2008). While *C.oppositophila* seems to be a high-elevation species, further work that examines the distribution of Papuan frog-biting midges across the altitudinal gradient is necessary to understand the elevational distribution of these species. Overall, we are just starting to understand the Papuan fauna of frog-biting midges and systematic trapping for specimens from this group using sound-baited traps, for instance, will undoubtedly reveal interesting ecological patterns and many additional species than those currently described.

## Supplementary Material

XML Treatment for Corethrella oppositophila
